# Structure-Activity Relationships of Bioengineered Heparin/Heparan Sulfates Produced in Different Bioreactors

**DOI:** 10.3390/molecules22050806

**Published:** 2017-05-15

**Authors:** Ha Na Kim, John M. Whitelock, Megan S. Lord

**Affiliations:** Graduate School of Biomedical Engineering, University of New South Wales, Sydney, NSW 2052, Australia; h.n.kim@unsw.edu.au (H.N.K.); j.whitelock@unsw.edu.au (J.M.W.)

**Keywords:** heparin, heparan sulfate, serglycin, proteoglycan, recombinant expression, bioreactor

## Abstract

Heparin and heparan sulfate are structurally-related carbohydrates with therapeutic applications in anticoagulation, drug delivery, and regenerative medicine. This study explored the effect of different bioreactor conditions on the production of heparin/heparan sulfate chains via the recombinant expression of serglycin in mammalian cells. Tissue culture flasks and continuously-stirred tank reactors promoted the production of serglycin decorated with heparin/heparan sulfate, as well as chondroitin sulfate, while the serglycin secreted by cells in the tissue culture flasks produced more highly-sulfated heparin/heparan sulfate chains. The serglycin produced in tissue culture flasks was effective in binding and signaling fibroblast growth factor 2, indicating the utility of this molecule in drug delivery and regenerative medicine applications in addition to its well-known anticoagulant activity.

## 1. Introduction

Heparin is used clinically as an anticoagulant due to its ability to bind anti-thrombin and modulate downstream events in the clotting cascade [1,2]. The large market for clinical heparin, including more than 300,000 doses used per day in the US [3,4], has also enabled researchers to explore its therapeutic application to reduce the thrombogenecity of materials and deliver growth factors for tissue repair [5]. Heparan sulfate is structurally similar to heparin with both being linear polysaccharides composed of repeating disaccharides of hexuronic acid and glucosamine. Heparin contains a higher degree of sulfation than heparan sulfate [6] with, on average, 2.7 sulfate groups per disaccharide, whereas heparan sulfate contains at least one sulfate group per disaccharide [7].

Heparin is only known to be expressed by mast cells in tissues that are in direct contact with the environment, including lung, skin, and intestine, and decorates the protein core of a single intracellular proteoglycan, serglycin [8,9,10,11]. Thus, the biological function of heparin is unlikely to be the prevention of blood coagulation. Heparan sulfate, however, is ubiquitous on the cell surface and in the extracellular matrix of tissues and decorates the protein core of many cell surface, including syndecans and glypicans, and extracellular matrix, including perlecan, agrin, and type XVIII collagen, proteoglycans and displays tissue-specific sulfation patterns [12,13]. These structural differences account for tissue-specific activities of heparan sulfates in modulating cellular interactions, as well as the binding and activity of enzymes, growth factors and extracellular matrix proteins [14,15]. Thus, there is growing interest in the therapeutic application of heparan sulfates for selective biological activities.

Both heparin and heparan sulfate isolated from tissues vary in composition and sequence between sources due to their synthesis via a non-template-driven process involving the timed activity of approximately twenty enzymes in the Golgi [16], although the regulators of the expression of these enzymes are not fully understood. These enzymes are involved in chain initiation, elongation, epimerization, and sulfation. While this structural heterogeneity provides an opportunity to fine-tune the biological activity for particular applications, the precise identification of structure-function relationships has been challenging [17]. However, certain structural features are known to be required for highly-specific interactions, such as a pentasaccharide structure containing an 3-*O*-sulfated glucosamine for binding to anti-thrombin III [18], whereas other structures are less specific, such as a contiguous string of highly-sulfated disaccharides for binding to fibroblast growth factor (FGF) and downstream growth factor activation [19].

Heparin is sourced predominantly from animal tissues, particularly porcine intestinal mucosa and, to a lesser extent, bovine lung tissues due to concerns over bovine spongiform encephalopathy contamination [20]. Commercially-available heparan sulfates are synthesized as byproducts of heparin production or by selective de-sulfation of heparin [21,22]. The production of heparan sulfate libraries from tissues is time consuming and technically challenging [23]. The growing demand for heparin and heparan sulfates for clinical applications has led researchers to explore alternative methods of production including chemoenzymatic synthesis [24], chemical synthesis [25], sulfation of polysaccharides [26], and metabolic engineering [27]. A bioengineered heparin-like heparan sulfate was recently reported by the authors by expressing serglycin in mammalian cells [1]. This recombinant serglycin was decorated with chondroitin/dermatan sulfate in addition to heparin/heparan sulfate chains [1,28] similar to serglycin isolated from natural sources where it has been shown to be decorated with multiple types of glycosaminoglycan chains covalently attached to its eight glycosaminoglycan attachment sites [8,9,10,11].

The aim of this study was to explore the effect of different bioreactor conditions on the yield, structure, and activity of heparin/heparan sulfates produced by expressing serglycin in mammalian cells. Bioreactors, including tissue culture flasks, continuously-stirred tank reactors (CSTR), and shaker flasks, were investigated as each of these have been used for commercial scale production of bioactives [29]. Different bioreactors and culture conditions were found to change the structure of the heparin/heparan sulfate chains produced by the cells with the serglycin produced being effective at binding and signaling FGF-2. This supports the use of these bioreactors and our approach to produce heparin/heparan sulfates for use in the clinic as an anticoagulant, as well as future uses in drug delivery and regenerative medicine applications.

## 2. Results

### 2.1. The Effect of Different Bioreactors on Serglycin Production

**Figure 1:**
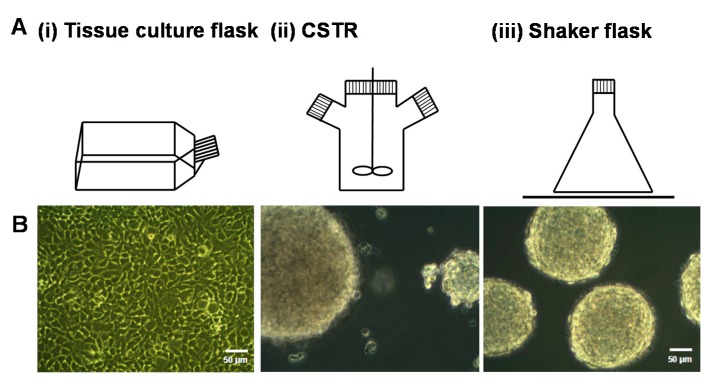
(**A**) Schematic of different bioreactors used to culture the HEK-293 cells expressing serglycin including (**i**) tissue culture flasks, (**ii**) continuously stirred tank reactors (CSTR), and (**iii**) shaker flasks; and (**B**) phase contrast images of cells after three days of culture in the different bioreactor conditions. The scale bar represents 50 μm.

HEK-293 cells expressing serglycin were cultured for three days in different bioreactors, including batch culture in tissue culture flasks, CSTR, and shaker flasks (Figure 1A). The morphology of cells after three days in culture was analyzed by phase contrast microscopy (Figure 1B). Cells cultured in the tissue culture flasks formed a confluent monolayer of cells with the characteristic polygonal morphology of adherent cells (Figure 1B(i)). In contrast, aggregated spheroids of cells were found in both the CSTR and shaker flasks (Figure 1B(ii),(iii)). Cells cultured in the CSTR were stirred at 100 rpm, which produced aggregates in the size range 50–300 μm (Figure 1B(ii)). Cells cultured in the shaker flasks were subjected to constant agitation using an orbital shaker operated at 80 rpm, which induced the formation of uniform cell spheroids that ranged in size from 180–200 μm (Figure 1B(iii)).

**Figure 2:**
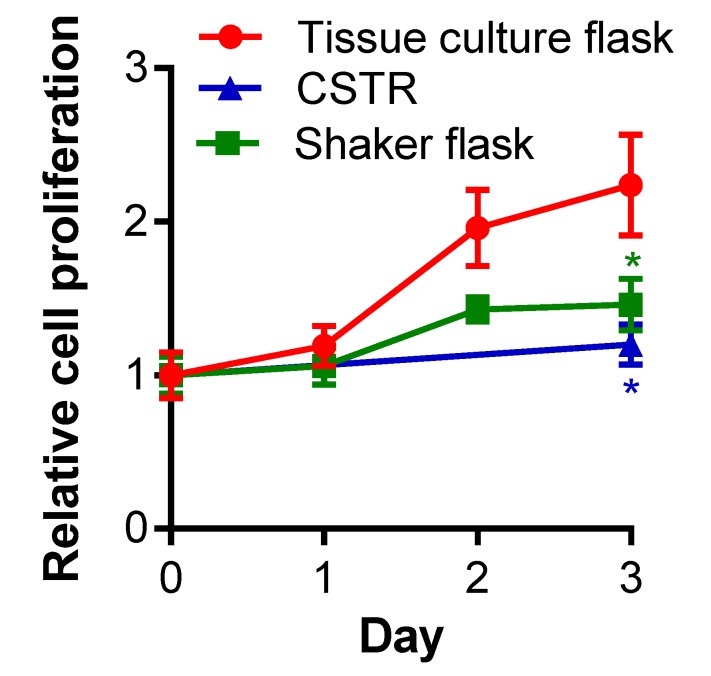
The relative number of cells measured over three days in the different bioreactors, including tissue culture flasks, CSTR, and shaker flasks. Data are presented as means ± standard deviation (*n* = 3). * indicates significant differences (*p* < 0.05) compared to tissue culture flasks at day 3 analyzed by one-way ANOVA.

The influence of the different bioreactors on cell proliferation was analyzed over three days (Figure 2). Cells cultured in the tissue culture flasks supported the highest level of cell proliferation. Both the CSTR and shaker flasks supported significantly reduced (*p* < 0.05) cell proliferation compared to the cultures in the tissue culture flasks. The CSTR and shaker flasks induced the formation of spheroid cultures that appeared to have reduced the proliferation of the cells.

**Figure 3:**
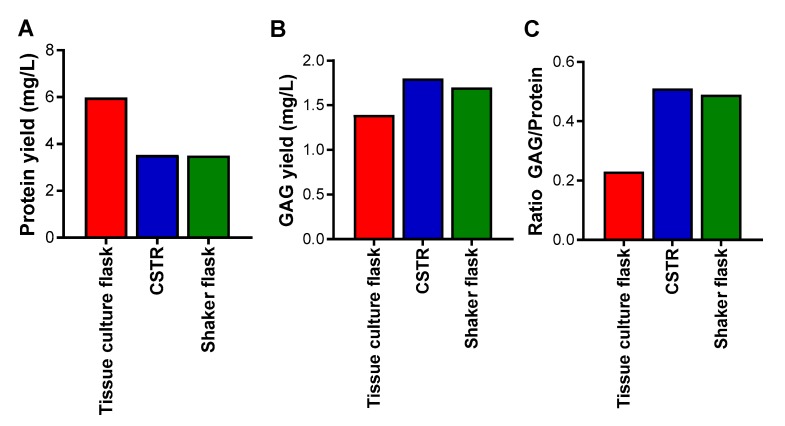
Yield of (**A**) protein; (**B**) glycosaminoglycan (GAG); and (**C**) the ratio of GAG to protein from HEK-293 cells expressing serglycin cultured in different bioreactors over three days and purified by anion exchange chromatography. Protein concentration was measured by Coomassie protein assay and GAG concentration was measured by Dimethylmethylene Blue (DMMB) assay.

The influence of the different bioreactors on the cells was also analyzed in terms of yield of proteins and glycosaminoglycans after enrichment of the conditioned medium by anion exchange chromatography for proteoglycans, of which the major proteoglycan produced by these cells was serglycin. The highest yield of proteins was obtained from the tissue culture flasks, followed by the CSTR, and then the shaker flasks, which demonstrated the lowest yield (Figure 3A). The highest yield of glycosaminoglycans was obtained from the CSTR and shaker flasks, and the lowest yield of glycosaminoglycans was obtained from the tissue culture flasks (Figure 3B). Analysis of the ratio of glycosaminoglycan to protein yields indicated that the CSTR and shaker flasks produced the highest amount, and the tissue culture flasks the least (Figure 3C). These data suggest that the spheroid cultures reduced cell proliferation and encouraged glycosaminoglycan decoration of the serglycin protein core to a greater extent than tissue culture flasks that encouraged cell proliferation and protein production.

**Figure 4:**
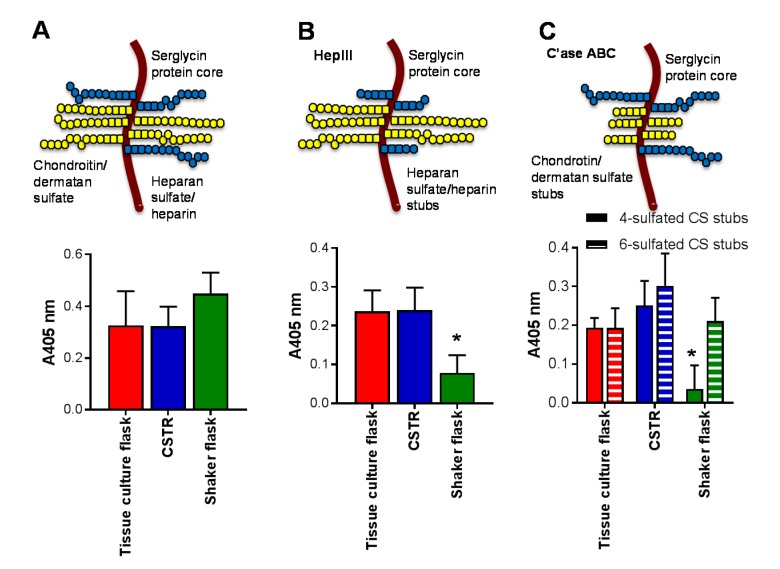
The effect of bioreactors on the production of serglycin, heparin/heparan sulfate and chondroitin sulfate. The schematic indicates the structure of serglycin with eight glycosaminoglycan attachment sites that can be decorated with either chondroitin/dermatan sulfate or heparin/heparan sulfate chains. The effect of glycosaminoglycan lyase digestion on the glycosaminoglycan chains are indicated in panels (**B**,**C**) with HepIII removing heparin/heparan sulfate chains to reveal a single stub structure and chondroitinase ABC (C’ase ABC) removing chondroitin/dermatan sulfate chains to reveal a stub structure. ELISA for the presence of (**A**) serglycin core protein; (**B**) heparin/heparan sulfate stubs detected using anti-heparan sulfate/heparin-stub antibody clone 3G10 following HepIII digestion, and (**C**) chondroitin sulfate stubs detected using anti-4-sulfated chondroitin sulfate stub antibody clone 2B6 and anti-6-sulfated chondroitin sulfate stub antibody clone 3B3 following C’ase ABC digestion. Data are presented as means ± standard deviation (*n* = 3). * indicates significant differences (*p* < 0.05) compared to tissue culture flasks analyzed by one-way ANOVA.

The effect of bioreactors on serglycin production and glycosaminoglycan decoration was also explored. There was no difference on the level of serglycin produced by cells in each of the bioreactors (Figure 4A). Tissue culture flasks and the CSTR produced the same level of heparin/heparan sulfate chains as detected by the presence of the HS stub structure that is demonstrated following heparinase III (HepIII) digestion and recognized by the monoclonal antibody 3G10 (Figure 4B). In contrast, the shaker flasks significantly reduced (*p* < 0.05) the level of heparin/heparan sulfate chains (Figure 4B). Unlike heparin/heparan sulfate, which has one stub structure, chondroitin sulfate (CS) has multiple stub structures, following chondroitinase (C’ase) ABC digestion, including the 4- or 6-sulfated stub that can be detected with monoclonal antibodies 2B6 or 3B3, respectively (Figure 4C). While the level of chondroitin sulfate chains with 6-sulfated stub structures was not altered in any of the bioreactors analyzed, the level of 4-sulfated stub structures was significantly reduced in the shaker flasks compared to the tissue culture flask and CSTR (Figure 4C).

**Figure 5:**
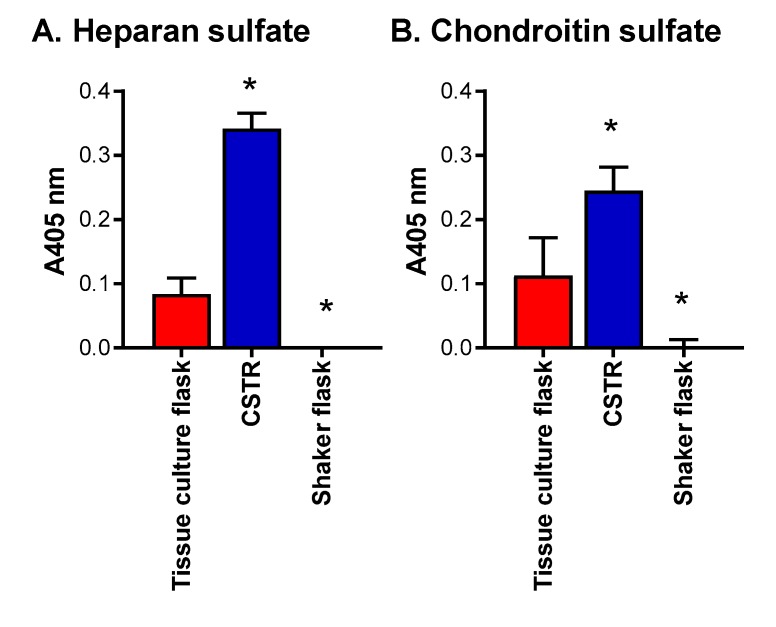
Effect of bioreactors on heparan and chondroitin sulfate structure. ELISA for the presence of (**A**) heparan sulfate chains detected using anti-heparan sulfate chain antibody clone 10E4 and (**B**) chondroitin sulfate chains detected using anti-chondroitin sulfate chain antibody clone CS-56. Data are presented as means ± standard deviation (*n* = 3). * indicates significant differences (*p* < 0.05) compared to batch cultures analyzed by one-way ANOVA.

The effect of the different bioreactors was further assessed by analyzing the sub-structure of the heparan and chondroitin sulfate chains produced by ELISA (Figure 5). Interestingly, the CSTR promoted the production of heparan sulfate chains containing N-acetylated glucosamine resides, as detected using the heparan sulfate chain antibody, to a significantly greater (*p* < 0.05) extent than either the tissue culture flasks or the shaker flasks (Figure 5A). Together with the data presented in Figure 4B, this indicated that the tissue culture flasks promoted heparan sulfate/heparin chains with a different structure to the CSTR as they exhibited the same level of heparan sulfate/heparin chains. It is speculated that the tissue culture flasks promoted heparan sulfate/heparin chains with sulfated disaccharides, as observed previously [1]. The shaker flasks facilitated minimal heparan sulfate/heparin chain production with no detectable heparan sulfate chains using the heparan sulfate chain antibody (Figure 5A). Both the tissue culture flasks and the CSTR supported the production of chondroitin sulfate containing type A and C disaccharides, while the shaker flask did not support the production of this type of chondroitin sulfate chain (Figure 5B). These data align with the level of 4-sulfated chondroitin sulfate stub structures detected in each of the cultures (Figure 4C).

### 2.2. The Effect of Different Culture Conditions on Serglycin Production

**Figure 6:**
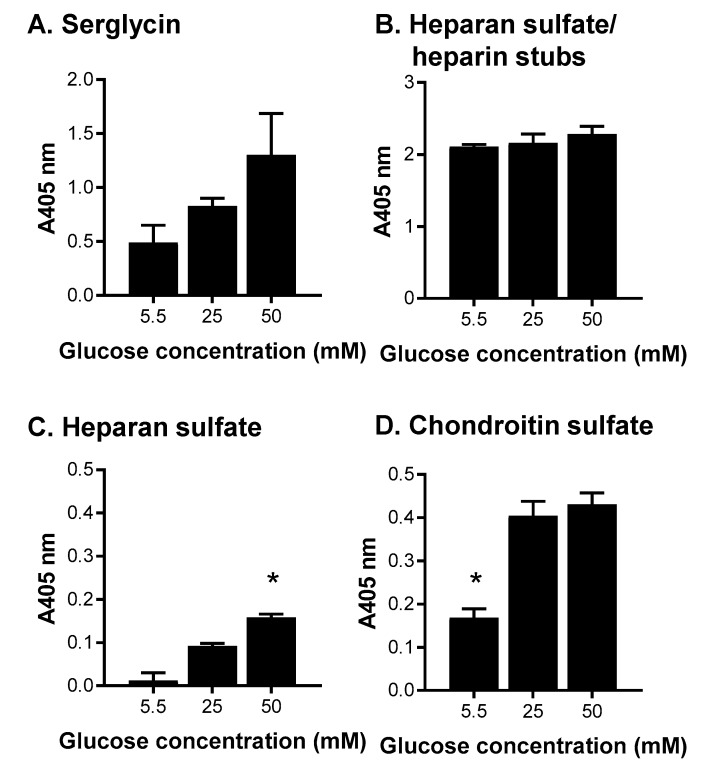
The effects of altering glucose concentrations in media for the production of serglycin, heparan sulfate/heparin and chondroitin sulfate. ELISA for the presence of (**A**) serglycin was detected using a polyclonal anti-serglycin antibody; (**B**) heparan sulfate/heparin stubs were detected using anti-heparan sulfate stub antibody clone 3G10 following HepIII digestion; (**C**) heparan sulfate chains were detected using anti-heparan sulfate antibody clone 10E4; and (**D**) chondroitin sulfate chains were detected using anti- chondroitin sulfate chain antibody clone CS-56. Data are presented as means ± standard deviation (*n* = 3). * indicates significant differences (*p* < 0.05) compared to 25 mM glucose analyzed by one-way ANOVA.

As the tissue culture flasks promoted the production of heparan sulfate/heparin, this type of bioreactor was explored further over a seven-day batch culture with HEK-293 cells expressing serglycin. Cells were cultured in three different glucose concentrations of 5.5, 25, and 50 mM as a previous study where the cultures that were passaged weekly and fed every three days indicated that the level of glucose in the medium affected heparin/heparan sulfate production [1]. The influence of glucose concentration on the cells was analyzed in terms of serglycin production and glycosaminoglycan decoration after enrichment of the conditioned medium by anion exchange chromatography for serglycin (Figure 6). Increasing the level of glucose in the medium increased the level of serglycin produced (Figure 6A). There was, however, no difference in the level of heparan sulfate/heparin stubs produced by cells in each of the conditions (Figure 6B) and only baseline levels of heparan sulfate chains containing *N*-acetylated glucosamine residues detected in each condition (Figure 6C). It is speculated that cells grown in tissue culture flasks for extended periods, regardless of glucose concentration, promoted heparan sulfate/heparin chains with sulfated disaccharides, as observed previously [1]. In contrast, cells cultured in medium containing 5.5 mM glucose produced significantly (*p* < 0.05) lower levels of chondroitin sulfate containing type A and C disaccharides compared to cells cultured in medium containing either 25 or 50 mM glucose (Figure 6D).

### 2.3. The Effect of Serglycin Glycosaminoglycan Decoration on Growth Factor Binding and Signaling

**Figure 7:**
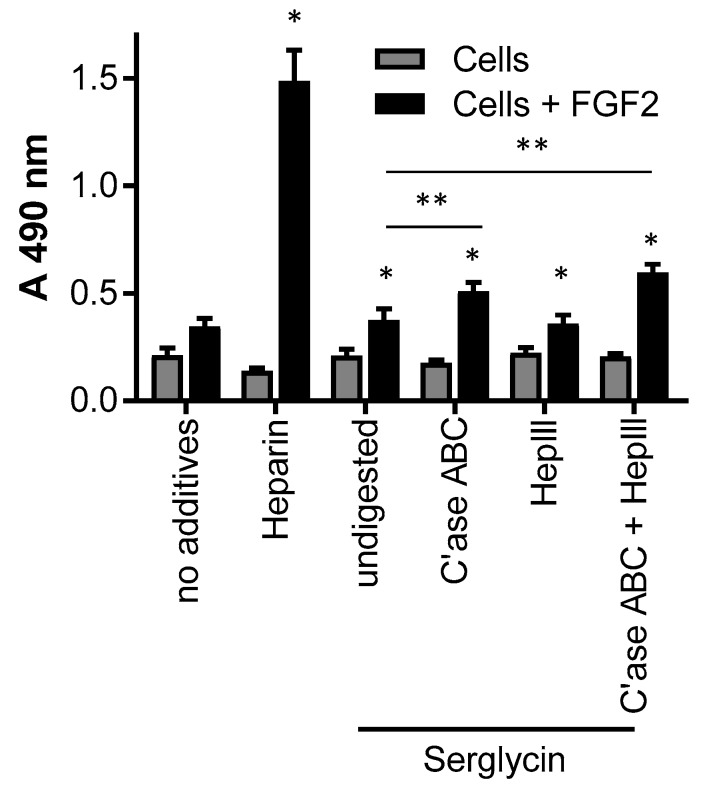
Activity of serglycin with heparin/heparan sulfate and chondroitin sulfate chains determined by the signaling of FGF receptor type 1c expressing BaF32 cells in the presence of FGF-2 as mesured by the MTS assay. Cells in the presence of FGF-2 and heparin were used as a control for the formation of active ternary complexes. Negative controls were cells in the presence of no additives, heparin, or FGF-2. Selected serglycin preparations were digested with either chondroitinase ABC, hepIII or both glycosaminoglycan lysases prior to the assay. Cell proliferation was measured after 72 h. * indicated significant differences (*p* < 0.05) within treatments for cells and FGF-2 compared to cells only as determined by a one-way ANOVA; ** indicated significant differences (*p* < 0.05) as determined by two-way ANOVA.

One of the major functions of the glycosaminoglycans, such as heparin/heparan sulfate, is to bind and signal growth factors. The production of proteoglycans capable of recapitulating this function in vivo is of therapeutic interest due to the relatively low abundance of naturally-occurring proteoglycans at sites of wound healing. Thus the binding and signaling of the mitogenic growth factor, FGF-2, was analyzed using the BaF32 cell assay. The BaF32 cells proliferate when biologically-active ternary complexes are formed between cell surface FGF receptors, FGF-2 and heparin/heparan sulfate. The positive control for the assay was the FGF receptor type 1c expressing cells exposed to heparin and FGF-2 as shown by the significant (*p* < 0.05) increase in absorbance compared to cells exposed to either heparin or FGF-2 (Figure 7). The addition of serglycin produced by cells cultured for seven days in medium containing 25 mM glucose, as this preparation contained both highly-sulfated heparan sulfate/heparin and chondroitin sulfate, bound and signaled FGF-2 as shown by the significant (*p* < 0.05) increase in cell proliferation compared to cells only exposed to serglycin. Digestion of the chondroitin sulfate chains that decorated serglycin resulted in significantly (*p* < 0.05) greater proliferation of the cells when exposed to FGF-2 compared to cells exposed to undigested serglycin and FGF-2. These data indicated that removal of the chondroitin sulfate chains increased the ability of the heparin/heparan sulfate chains that decorated serglycin to bind and signal FGF-2. Digestion of the heparin/heparan sulfate chains that decorated serglycin resulted in no significant difference in the proliferation of the cells when exposed to FGF-2 compared to cells exposed to undigested serglycin and FGF-2. Digestion of the heparin/heparan sulfate and chondroitin sulfate chains that decorated serglycin resulted in a significant (*p* < 0.05) increase in the proliferation of the cells when exposed to FGF-2 compared to cells exposed to undigested serglycin and FGF-2. Together these data suggested that the heparin/heparan sulfate chains released from the serglycin core protein by HepIII digestion were able to form active complexes with FGF-2 and FGF receptor type 1c.

## 3. Discussion

This study explored the production of recombinant serglycin in different bioreactors including tissue culture flasks, CSTR, and shaker flasks. Bioreactors that maintain cells in a suspension culture are widely used in industry enabling scale-up of bioactive production [29]. The HEK-293 cells used in this study that had been transfected to stably express serglycin are an adherent cell line, so it was of interest to subject them to the CSTR and shaker flask bioreactors that did not provide conditions for cell adhesion and explore their ability to proliferate and express serglycin. Interestingly, the cells cultured in these bioreactors proliferated, albeit at reduced levels compared to the cells grown in tissue culture flasks. In addition, the cells in both the CSTR and shaker flasks formed spheroids similar to what can be achieved with packed bed bioreactors, however, without an adhesive bead matrix to support cell adhesion [30]. The cell spheroids formed in the CSTR and shaker flasks indicated that the cells under these conditions, formed stable cell-cell contacts providing a different microenvironment to cells grown in the tissue culture flasks.

The microenvironment of cells is known to affect the type and structure of glycosaminoglycans that decorate proteoglycans leading to tissue-specific sulfation patterns [12,13]. The authors recently reported that mast cells cultured in different microenvironments change the type of glycosaminoglycans that they produce [31]. Thus, it was not surprising to discover in this study that the type of bioreactor used to produce proteoglycans altered the production of heparin/heparan and chondroitin sulfate chains. Interestingly, while there were differences in the extent and type of glycosaminoglycans produced, there was no change in the level of serglycin produced in each of the bioreactors. It was interesting to note that while the CSTR and shaker flask bioreactors both induced cell spheroid cultures, albeit of different sized spheroids, the cells produced a similar level of glycosaminoglycan decoration of the protein cores, but with different structures. This finding further supports the concept that the microenvironment plays a key role in determining the type and structure of glycosaminoglycans.

The level of glucose in the culture medium has been reported to affect cell proliferation [32], as well as protein production and glycosylation [33,34,35,36]. A comparison of the results of this study and a previous study by the authors indicated that changes in the method of culture in tissue culture flasks is sufficient to alter both serglycin protein core production and the type of glycosaminoglycans [1]. Previously, the cells were cultured for seven days in tissue culture flasks in medium containing 5.5, 25, or 50 mM glucose, passaged weekly and fed every three days. These conditions indicated that the level of glucose in the medium affected both serglycin and heparin/heparan sulfate production [1]. In contrast, this study, cells were grown for three days in tissue culture flasks in medium containing 5.5, 25, or 50 mM glucose prior to passaging, demonstrated that the level of glucose in the medium also affected chondroitin sulfate production.

As the recombinant serglycin produced in this study was decorated with heparin/heparan sulfate chains, it was of interest to determine its ability to bind and signal growth factors. Heparin/heparan sulfate chains are known to bind FGF-2 involving regions of high sulfation called S-domains [19,37]. Thus the serglycin preparation containing sulfated heparan sulfate/heparin and chondroitin sulfate was explored for FGF-2 signaling. This study found that the serglycin bound and signaled FGF-2 through its heparin/heparan sulfate chains both when attached to the serglycin protein core and following HepIII digestion. HepIII depolymerizes heparan sulfate by elimination of hexuronic acids acting next to *N*-sulfated or *N*-acetated residues without sulfation, or with low levels of O-sulfation [38]. Thus HepIII has a limited ability to depolymerize heparin. The heparin/heparan sulfate chains attached to serglycin used in this assay were found to contain highly-sulfated disaccharides, thus, treatment with HepIII would have released heparin/highly-sulfated heparan sulfated oligomers accounting for the FGF-2 activity of the heparin/heparan sulfate chains in solution in addition to when presented on the protein core of serglycin. While chondroitin sulfate is not involved in FGF-2 signaling [39], removal of the chondroitin sulfate chains from serglycin in this study was able to enhance the binding and signaling of the heparin/heparan sulfate bound to serglycin. This is likely due to the close proximity of the eight glycosaminoglycan attachment sites within the central region of the serglycin protein core. Removal of the chondroitin sulfate chains from the protein core of serglycin is likely to have enabled increased flexibility of the heparin/heparan sulfate chains attached to serglycin. A similar phenomenon has been observed for perlecan decorated with both heparan and chondroitin sulfate [40].

In conclusion, this study demonstrated that different bioreactors, including tissue culture flasks, CSTRs, and shaker flasks, in addition to different levels of glucose in the media, produced unique microenvironments that differentially decorated serglycin with heparin/heparan sulfate, as well as chondroitin sulfate. The serglycin produced bound and signaled FGF 2 via its heparin/heparan sulfate chains both when presented as a proteoglycan or as isolated glycosaminoglycan chains. These data demonstrate that the recombinant expression of serglycin is a promising approach for the production of tailored glycosaminoglycan structures with broad applications in drug delivery and regenerative medicine.

## 4. Materials and Methods

### 4.1. Culture of Mammalian Cells Expressing Serglycin

Human embryonic kidney (HEK-293) cell were transfected to stably express serglycin, as previously described [1]. Cells were maintained with DMEM culture medium containing 25 mM glucose, 10% (*v*/*v*) fetal bovine serum (FBS) and 100 μg/mL penicillin and streptomycin at 37 °C, 5% CO_2_ in a humidified incubator. Cells seeded at 2 × 10^5^ cells/mL were cultured in standard T75 culture flasks (reactor volume of 10 mL), a continuously-stirred tank reactor (CSTR) operated at 100 rpm, or a conical shaker flask on an orbital shaker operated at 80 rpm, each with a reactor volume of 250 mL. The speed of rotation of the CSTR and shaker flasks was determined with reference to previous studies using suspension cells [31]. Each of the bioreactors was operated in batch mode over three days. Additionally, cells were cultured in DMEM culture medium containing 5.5, 25, or 50 mM glucose, 10% (*v*/*v*) FBS and 100 μg/mL penicillin and streptomycin at 37 °C, 5% CO_2_ in a humidified incubator in standard T75 culture flasks in batch mode for seven days.

### 4.2. Cell Proliferation Assay

At each time point 50 µL of cell suspension was removed from the CSTR and shaker flasks and the number of viable cells analyzed using a hemocytometer and the trypan blue exclusion dye. For the tissue culture flasks, cell proliferation was analyzed by setting up parallel conditions in a 96 well tissue culture plate seeded with 2 × 10^5^ cells/mL and the number of cells at each time point was analyzed by the MTS assay using Cell Titer 96 Aqueous One Solution Reagent (Promega, Madison, WI, USA) and absorbance was measured at 490 nm.

### 4.3. Isolation of Serglycin

Serglycin was isolated from the conditioned medium by anion exchange chromatography. The diethylaminoethyl (DEAE) column was equilibrated at 1 mL/min with running buffer (250 mM NaCl, 20 mM Tris, 10 mM EDTA, pH 7.5) before the addition of medium conditioned by cells and the baseline absorbance was re-established with running buffer. Serglycin was eluted using an eluting buffer (1 M NaCl, 20 mM Tris, 10 mM EDTA, pH 7.5) and concentrated. Serglycin-enriched fractions were subsequently concentrated and analyzed for protein concentration using a Coomassie Blue protein assay (Thermo Scientific, Scoresby, Australia). Glycosaminoglycan concentration was determined using the Dimethylmethylene Blue assay (DMMB) as previously described [41].

### 4.4. Glycosaminoglycan Digestion

Samples were digested with 50 mU/mL proteinase-free chondroitinase (C’ase) ABC (EC 4.2.2.4) purified from *Proteus vulgaris* in 0.1 M Tris acetate, pH 8 at 37 °C for 16 h to confirm the presence of CS/DS. Samples were digested with 10 mU/mL of heparinase (Hep) III (EC 4.2.2.8) purified from *Flavobacterium heparinum* (Seikagaku Corp., Tokyo, Japan) diluted in 10 mM Tris-HCl, pH 7.4 at 37 °C for 16 h to determine the presence of heparin/HS.

### 4.5. ELISA

Serglycin-enriched samples (10 μg based on Coomassie protein assay) with and without glycosaminoglycan lyase digestion were coated onto high-binding 96-well ELISA plates (Greiner, Frickenhausen, Germany) for 2 h at 25 °C. Wells were rinsed twice with Dulbecco’s phosphate-buffered saline, pH 7.4 (DPBS) followed by blocking with 0.1% (*w*/*v*) casein in DPBS for 1 h at 25 °C. Wells were rinsed twice with DPBS with 1% (*v*/*v*) Tween 20 (PBST) followed by incubation with primary antibodies diluted in 0.1% (*w*/*v*) casein in DPBS for 2 h at 25 °C. Primary antibodies used included a rabbit polyclonal anti-serglycin antibody (ascites 1:5000), mouse monoclonal anti-4-sulfated chondroitin sulfate stub antibody (clone 2B6, gift from Prof Bruce Caterson, Cardiff University, conditioned medium 1:1000), mouse monoclonal anti-6-sulfated chondroitin sulfate stub antibody (clone 3B3, gift from Prof Bruce Caterson, Cardiff University, conditioned medium 1:1000), mouse monoclonal anti-heparan sulfate stub antibody (clone 3G10, Seikagaku, 1 µg/mL), mouse monoclonal anti-chondroitin sulfate type A and C antibody (clone CS-56, ascites 1:2500), and mouse monoclonal anti-heparan sulfate antibody (clone 10E4, Seikagaku Corp., 1 µg/mL). Wells were rinsed twice with PBST, followed by incubation with biotinylated secondary antibodies (1:1000) diluted in 0.1% (*w*/*v*) casein in DPBS for 1 h at 25 °C, rinsed twice again with PBST, and then incubated with streptavidin-HRP (1:500) for 30 min at 25 °C. Binding of the antibodies to the samples was detected using the colorimetric substrate, 2,2-azinodi-(3-ethylbenzthiazoline sulfonic acid), and absorbance was measured at 405 nm.

### 4.6. Growth Factor Binding and Signaling of FGF-2

BaF32 cells were derived from an IL-3-dependent and heparan sulfate proteoglycan-deficient myeloid B cell line that has been stably transfected with fibroblast growth factor receptor (FGFR) 1c [42]. BaF32 cells represent a model system developed to identify heparin/heparan sulfate structures that interact with FGFs and their receptors. The readout of this assay is cell proliferation which indicated the formation of ternary complexes on the cell surface between heparin/heparan sulfate, FGF-2 and FGFR1c. BaF32 cells were maintained in RPMI 1640 medium containing 10% (*v*/*v*) FBS, 10% (*v*/*v*) WEHI-3BD conditioned medium, 100 µg/mL penicillin, and streptomycin. WEHI-3BD cells were maintained in RPMI 1640 medium supplemented with 2 g/L sodium bicarbonate, 10% (*v*/*v*) FBS, 100 µg/mL penicillin, and streptomycin, and the conditioned medium was collected three times per week and stored at −20 °C until it was required. For the mitogenic assays, the BaF32 cells were transferred into IL-3 depleted medium for 24 h prior to experimentation and seeded into 96-well plates at a density of 2 × 10^4^ cells/well in the presence of medium only, 120 nM heparin, 0.5 µg/mL serglycin either in the presence or absence of 0.03 nM FGF-2. To analyze the role of the different glycosaminoglycans that decorated recombinant serglycin, serglycin was also treated with C’ase ABC and/or HepIII prior to use in the assay. Cells exposed to heparin and FGF-2 were used as a positive control for the assay, as this combination is known to induce cell proliferation, while cells exposed to each of the treatments in the absence of FGF-2 were used as a negative control. Background absorbance readings were also obtained for each of the treatments in the absence of cells. Cells were incubated for 72 h in 5% CO_2_ at 37 °C, and the number of cells present was assessed using the MTS assay. The MTS reagent (Promega, Madison, WI, USA) was added to the cell cultures 6 h prior to measurement of the absorbance at 490 nm.
